# Epidemiology and Risk Factors of Cervical Spine Injury during Heating Season in the Patients with Cervical Trauma: A Cross-Sectional Study

**DOI:** 10.1371/journal.pone.0078358

**Published:** 2013-11-04

**Authors:** Sidong Yang, Wenyuan Ding, Dalong Yang, Tixin Gu, Feng Zhang, Di Zhang, Yapeng Sun, Lei Ma, Yanli Song

**Affiliations:** 1 Department of Spine Surgery, The Third Hospital of Hebei Medical University, Shijiazhuang, Hebei, China; 2 Department of VIP Ward, the First Hospital of Hebei Medical University, Shijiazhuang, Hebei, China; 3 Department of Rehabilitation Medicine, The Third Hospital of Hebei Medical University, Shijiazhuang, Hebei, China; 4 Hebei Provincial Key Laboratory of Orthopedic Biomechanics, Shijiazhuang, Hebei, China; The University of Tokyo, Japan

## Abstract

**Purpose:**

The purpose of this study was to describe the epidemiology of cervical spine injury in the patients with cervical trauma and analyze its associated risk factors during the special heating season in North China.

**Methods:**

This cross-sectional study investigated predictors for cervical spine injury in cervical trauma patients using retrospectively collected data of Hebei Provincial Orthopaedic Hospital from 11/2011 to 02/2012, and 11/2012 to 02/2013. Binary logistic regression analysis was used to determine risk factors for cervical fractures/dislocations or cord injury.

**Results:**

A total of 106 patients were admitted into this study. Of all, 34 patients (32.1%) were treated from 11/2011 to 02/2012 and 72 patients (67.9%) from 11/2012 to 02/2013. The mean age was 41.9±13.3 years old; 85 patients (80.2%) were male and 82 (77.4%) from rural areas. Eighty patients (75.5%) were caused by fall including 45 (42.5%) by severe fall (>2 m). Sixty-five patients (61.3%) of all suffered injuries to other body regions and 32 (30.2%) got head injury. Thirty-one patients (29.2%) sustained cervical cord injury with cervical fractures/dislocations. Twenty-six (83.9%) of cervical cord injury patients were from rural areas and 24 (77.4%) of those resulted from fall including 15 (48.4%) from severe fall (>2 m). Logistic regression displayed that age (OR, 1.47; 95% CI, 1.05–2.07), head injury (OR, 5.63; 95% CI, 2.23–14.26), were risk factors for cervical cord injury and snowing (OR, 8.25; 95% CI, 2.26–30.15) was a risk factor for cervical spine injury due to severe fall (>2 m).

**Conclusions:**

The elder male patients and patients with head trauma are high-risk population for cervical cord injury. As a seasonal factor, snowing during heating season is of note a risk factor for cervical spine injury resulting from severe fall (>2 m) in the patients with cervical trauma in North China.

## Introduction

Trauma causes 10% of death worldwide and is the leading cause of death for young people ranging from 5 to 44 years old in developed countries [1], [2]. For trauma-related spinal fractures, the reported annual incidence rate varies from 0.019% to 0.088% [3], [4], and for spinal cord injury, from 35 to 53 per million people [3]–[7]. Between 19% and 51% of cases of spinal trauma involve injuries to the cervical spine [8]. Patients with cervical spine injuries are a high-risk group, with the highest reported early mortality rate in spinal trauma, as these injuries may be associated with spinal cord injury. Therefore, it is urgent to find the risk factors as early predictors of spinal cord injury, so that essential treating measures will be timely taken to decrease early mortality.

However, epidemiology of spine injury and cord injury may be different from one to another, for its own specialty. Gupta A and Reeves B reported that Fijian spine injury and cord injury resulting from mango tree falls were an obvious seasonal scourge [9]. And this seasonal trend is likely to also exist in North China. There is a heating period annually between November and the next February in North China when fog and snow are common seen here and there. Cervical spine trauma and spinal cord injury seem to occur more often during this special period.

Statistical data from our hospital shows that almost one half of cervical cord injury appears during this period along the whole year of 2012. However, in recent years few studies have been focused on the special impact of misting or snowing on cervical spine injury or cord injury. Therefore, this retrospective cross-sectional study is focused on the epidemiology of cervical spine injury in the patients with cervical trauma and throws light upon snowing effect, in an effort to find the potential risk factors underlying cervical spine injury in the patients with cervical trauma in the heating season of North China.

## Patients and Methods

### Patients

This study included patients with cervical fracture with or without cervical cord injury, who were admitted into Trauma Emergency Centre or Department of Spinal Surgery in Hebei Provincial Orthopaedic Hospital in China between 11/2011 and 02/2012, 11/2012 and 02/2013. The inclusion criteria were plain radiographs in two planes, computed tomography scans and complete medical records. These criteria were met in 106 cases, which were therefore incorporated into this retrospective cross-sectional analysis.

### Methods

The current study was approved by Ethics Committee of the Third Hospital of Hebei Medical University, also known as Ethics Committee of Hebei Provincial Orthopedic Hospital. There is no need to provide informed consent since all the data were collected and analyzed anonymously. And it was approved by Ethics Committee of the Third Hospital of Hebei Medical University, also known as Ethics Committee of Hebei Provincial Orthopedic Hospital.

The analysis was focused on patient-related data including age at trauma incident, gender and regional distribution, injury mechanism (traffic accident, mild fall, severe fall), concurrent injuries and head injury. As snowing was obviously observed regarding its influence for spinal cord injury, it was also analyzed as a potential risk factor. For statistical analysis, SPSS 18.0 for windows was used to perform chi-square test and logistic regression. Values for p < 0.05 were regarded as significant.

## Results

Of the 111 patients initially identified, a total of 106 patients were admitted into this study and 5 were out for data deficiency. Of all, 34 patients were treated from 11/2011 to 02/2012 and 72 patients from 11/2012 to 02/2013. Average hospital stay was 13.3 days in total patients, and 13.6 days in cord-injury patients.

### Distribution of age, gender, and regions

**Figure 1:**
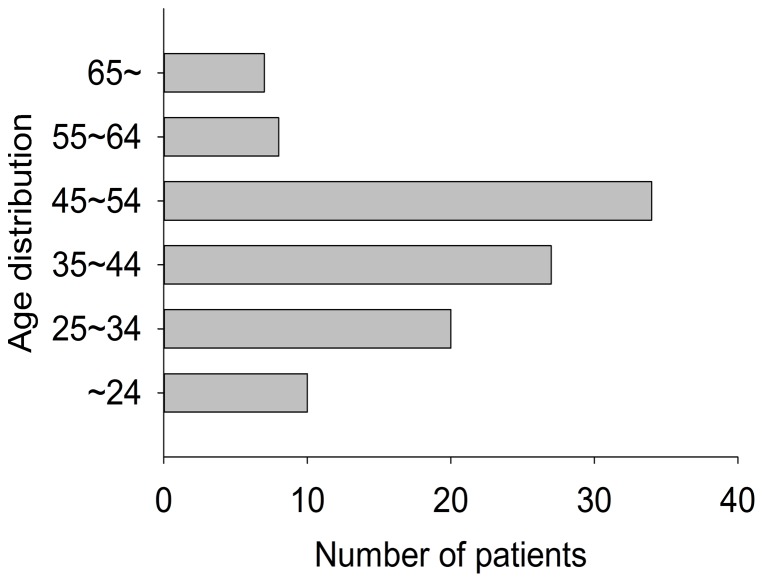
Age distribution of total patients.

**Figure 2:**
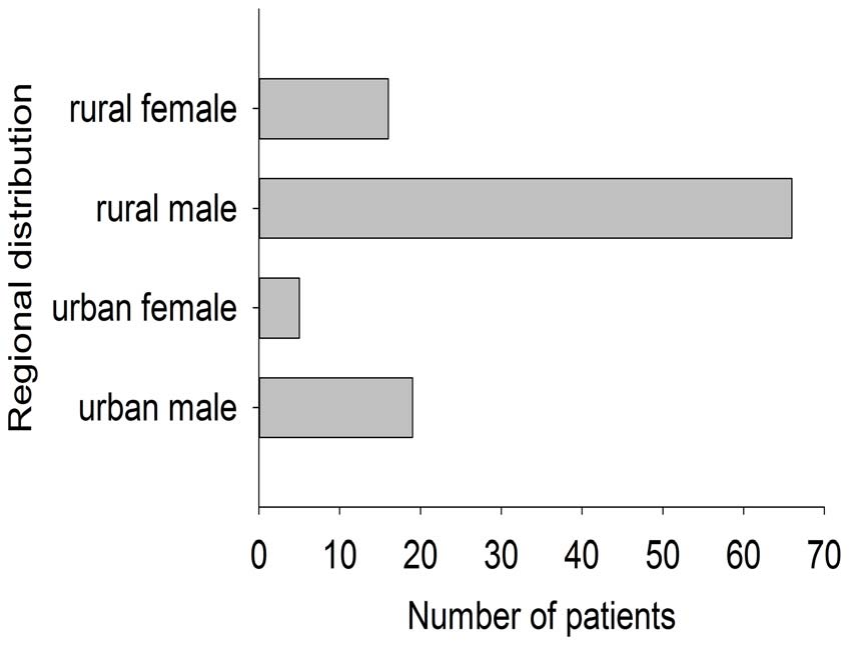
Regional distribution of total patients.

All patients were grouped into six categories according to their age (∼24; 25∼34; 35∼44; 45∼54; 55∼64; 65∼) as figure 1 showed. The mean age was 41.9±13.3 years old. Apparently, younger patient population ranging from 25 to 54 years old ranked the most. Two categories were grouped according to region (urban areas and rural areas). In China, the areas were grouped into 3 types according to nonagriculture population size including cities (>35000), towns (>2000) and countrysides (<2000). The population size is different due to local peculiarity in Hebei Province. For example, the population of the biggest city and the smallest city is 3 million and 35 thousand, respectively. Because the number of rural areas is much larger than that of urban areas, the total population size of rural areas is bigger than that of urban areas. In this study, urban areas include cities and towns, while rural areas mean countrysides. As displayed in figure 2, rural patients (n = 82, 77.4%) were the major patient population of cervical spine injury. Male patients (n = 85, 80.2%) took a majority of patient population both in urban areas and rural areas. Totally, distribution of age, gender, and regions released that young male labor from rural areas were main patient population responsible for cervical spine injury.

### Injury mechanism

**Figure 3:**
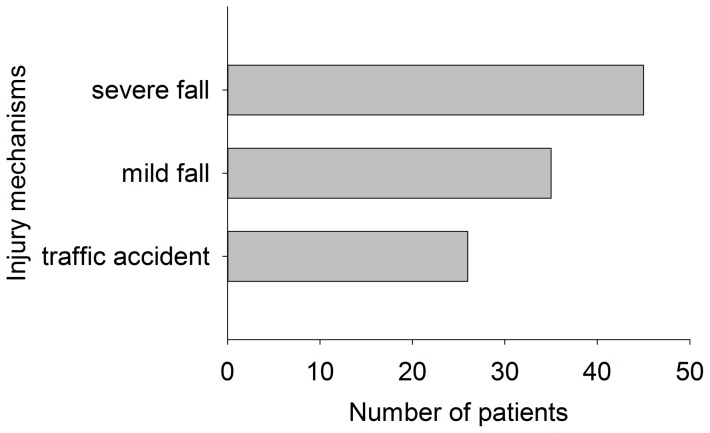
Distribution of injury mechanisms in total patients.

The injury mechanisms were categorized into traffic accident, mild fall, and severe fall (>2 m) according to patient case record. Twenty-six patients (24.5%) resulting from traffic accident took a minority of patient population. Eighty patients (75.5%) were caused by fall including 45 (42.5%) by severe fall (>2 m). Of note, severe fall (>2 m) ranked first regarding major causes of cervical spine injury as exhibited in figure 3.

### Concurrent injuries

**Figure 4:**
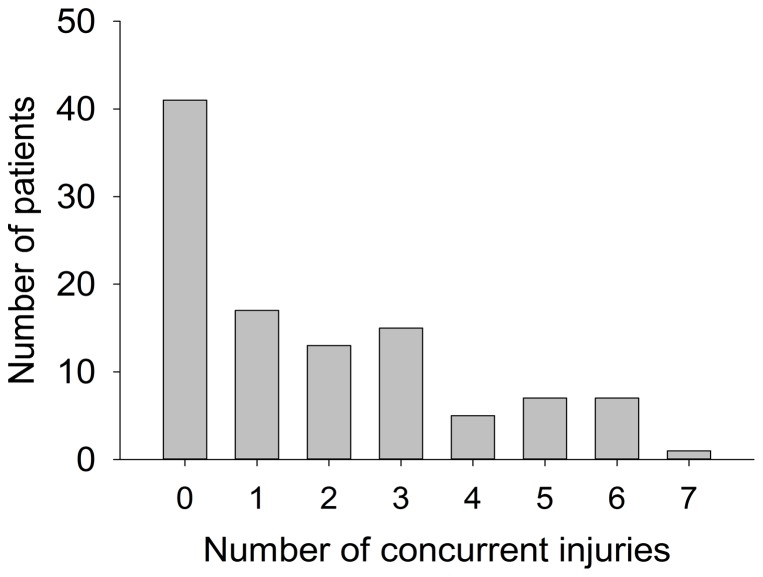
Distribution of concurrent injuries in total patients.

Concurrent injuries indicated that there were other body traumas except for cervical fractures/dislocations and cervical cord injury, such as thoracic and lumbar fractures/dislocations, head injury, pelvic fracture, limb fracture, organ injury [8], [10]. We counted the number of concurrent injuries according to injuries at different anatomic regions. For example, when encountering cervical injury with thoracic vertebrae fracture and rib fracture, we defined the number of concurrent injuries as 2. But for cervical injury with multiple thoracic vertebrae fractures, the number of concurrent injuries was considered as 1. For multiple organ injuries, we counted the number of injuried organs as concurrent injuries in total. In this study, the average number of concurrent injuries was 1.8. Sixty-five (61.3%) patients sustained injuries to other body regions and 30.2% (n = 32) got head injury, as shown in figure 4. And most of them were males from rural regions.

### Cervical cord injury

Thirty-one patients (29.2%) sustained cervical cord injury with cervical fractures/dislocations. 83.9% (n = 26) of cervical cord injury patients were from rural areas and 77.4% (n = 24) of those resulted from fall including 48.4% (n = 15) from severe fall (>2 m). Binary logistic regression revealed that age and head injury were effective predictors for cervical cord injury as table 1 showed. The logistic equation was logit P  =  –2.212 + 0.388*X_1_ + 1.729*X_2_, (X_1_ =  age, OR =  1.47; 95%CI, 1.05–2.07 ; X_2_ =  head injury, OR  =  5.63; 95%CI, 2.23–14.26). And the equation was statistically significant by Pearson Chi-Square Test (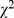 = 16.91, P<0.001).

**Table 1:** OR of age, gender, regional distribution, injury mechanisms and snowing.

	OR	95% CI	
**Age (yr)**			
∼24	1.00		reference
25∼34	3.00		(1.30, 5.94)
35∼44	4.50		(2.49, 6.25)
45∼54	3.75		(2.42, 5.63)
55∼64	5.40		(3.44, 8.67)
65∼	12.00		(7.94, 18.89)
**Gender**			
Male	0.79		(0.28, 2.19)
Female	1.00		reference
**Regional distribution**			
Urban area	1.00		reference
Rural area	1.76		(0.59, 5.24)
**Injury mechanism**			
Traffic accident	1.00		reference
Mild fall	0.94		(0.30, 2.97)
Severe fall(>2 m)	1.36		(0.47, 3.94)
**Snowing**			
Before snowing	1.00		reference
After snowing	8.25		(2.26, 30.15)

### Influence of snowing

There were two times of snowing between 11/2011 and 02/2012, and three between 11/2012 and 02/2013. Totally, there was no significant difference in the morbidity of cervical spine injury surrounding the snowing day. But snowing did make a significant difference in cervical spine injury patients caused by severe fall (>2 m) between the period of one week before snowing and the period of one week after snowing (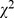 = 11.29, P = 0.001), just as table 2 displayed. The odds ratio (after snowing/before snowing) was 8.25, and 95% confidence interval (2.26, 30.15). We also compared patients caused by severe fall during one week before snowing to those during one week after snowing in the heating period alone from 11/2012 to 02/2013. As table 3 showed, snowing was proved to be a risk factor (P = 0.017, Fisher’s Exact Test). The odds ratio was 6.60 and 95% CI (1.53, 28.52).

**Table 2:** Patient population in total during one week before or after snowing.

Group	Severe fall(>2 m)	Others
Before snowing	5	22
After snowing*	15	8

*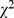 = 11.29, P = 0.001 compared with group (before snowing) by Pearson Chi-Square Test.

**Table 3:** Patient population during one week before snowing or after snowing from 2012/11 to 2013/02.

Group	Severe fall(>2 m)	Others
Before snowing	5	15
After snowing*	11	5

* P = 0.017 compared with group (before snowing) by Fisher’s Exact Test.

## Discussion

### Summary of Findings

We found that ORs for cervical cord injury increased along with increasing age of the patients, and with head injury. ORs for cervical fractures/dislocations were raised in patients with dangerous injury mechanism ( severe fall >2 m). Eighty-five (80.2%) patients with cervical fractures/dislocations were male. Eighty patients (75.5%) were caused by fall. Sixty-five patients (61.3%) of all suffered concomitant injuries and 30.2% (n = 32) got head injury. Thirty-one patients (29.2%) sustained cervical cord injury with cervical fractures/dislocations, and 48.4% (n = 15) of cord injury resulted from severe fall.

### Comparison With Other Studies

In this study, we found that increasing age was a risk factor for cervical cord injury, almost consistent with the previous study of Hasler RM et al [8]. However, they reported that ORs for cervical cord injury increased with increasing age in patients older than 35 years, different from the 25 years we reported. Furthermore, head injury in our study was found to be a useful predictor for cervical cord injury, contrary to what Hasler RM et al stated. Of note, the association of cervical spine injury and concomitant head trauma has been a controversial issue [8], [11]–[13]. Mulligan et al. detected cervical spine injury in 7% of patients with head, but without further significance test [14]. Smith et al. found 49% of the patients with cervical spine fractures sustained a concomitant brain injury [15]. The corresponding figure reported by Hasler RM et al. was only 24.7%,whereas ours was 30.2% between them.

As previously reported, high-energy traumas (traffic accident, severe fall, and so on) were more likely to be accompanied by associated injuries, such as head trauma or extremity fractures. [10] Conversely, isolated cervical fractures in the elderly were often caused by mild fall, which was sufficient enough to induce bone failure due to reduced bone mineral density, but failed to result in concomitant injuries. Additionally, No difference was found in age distribution between the patients sustaining concomitant injuries and those without concomitant injuries, contradictory to a previous report that associated injuries were on average six years younger than patients with an isolated spine fracture [10].

Concurrent injuries were a common finding in this study population. The most common concurrent injury was found to be head injury, which was in line with other investigations [10], [16], [17]. To common belief, head injury was believed predictive for cervical spine injury. Tian HL et al. [18] reported that cervical spine injury was associated at a significantly higher incidence with motorcycle accident-related head trauma as compared with non-motorcycle accident-related trauma (10.32% vs. 4.68%). In this study, head injury was also proved to be a predictive factor for cervical cord injury with a high incidence of 30.2%.

We revealed in the present study that 48.4% of cervical cord injury resulted from severe fall and 22.6% from traffic accidents. This was different from the report of Ho CH et al. [19], stating that motor vehicle crashes ranked the first place in causing spinal cord injury and the rates for falls only ranked 23.8% between the year 2000 and 2003. This may be attributed to our unique study design focusing on the heating season, different from their whole year study. Similar to the present study, Gupta A and Reeves B reported that Fijian spine injury and cord injury resulting from mango tree falls was an obvious seasonal scourge [9].

Severe fall has been associated to cause more spinal cord injuries than motor vehicle accidents [10]. Zargar et al. [20] from Iran, associated 22.4% of tree falls with spinal cord injuries and Mulford et al. [21] reported 16.3% of coconut tree falls resulted in spinal cord injuries. Neurological deficits were found in about 14–38% of all vertebral fractures [17], [22], and represented the most devastating consequence of spinal fractures. In our study neurological deficits were found in 29.2% of patients.

An obvious male gender trend and a rural region trend were observed for cervical spine injury, suggesting that rural men were more likely to sustain cervical trauma, either due to their professions or recreational activities. In rural areas, male population are main labor bearing the burden of supporting a family. That increases their outdoor activities accompanied by more exposure of risk factors. That is why the male gender in rural region were found at high risk for cervical spine injury.

The city which our hospital is located in is well representative of North China in terms of a typical winter cold climate. Therefore, there is a heating period annually between November and the next February in this city when fog and snow are more common seen than before. Snowing in this historical cross-sectional study, as a hypothetical impact factor, got statistically tested in its influence on cervical spine injury. The outcome displayed that the total morbidity of cervical spine injury was not increased after snowing. Nevertheless, cervical spine trauma caused by severe fall was significantly raised just one week after snowing. This phenomenon was unique during the special heating period in North China, and no similar study has reported this before. Overall, this cross-sectional study indicated that snowing did increase the risk for cervical spine injury resulting from severe fall during the heating season.

### Implications

This is a cross-sectional study on cervical spine injury and its predictors in cervical trauma patients. Although computed tomographic imaging of the cervical spine is part of common trauma protocols, this study laid emphasis on the prevention for cervical spine injury, and the careful protection and diagnostic imaging in these patients. Furthermore, in presumed non-polytraumatized patients or patients with less obviously recognizable injuries and dangerous injury mechanisms, protection and diagnostic imaging of the cervical spine are mandatory.

### Strengths and Weaknesses

Overall, this study was able to detect some interesting correlations that will be able to guide primary care physicians in their initial diagnostic work up. However, the retrospective nature of our work goes along with limitations, the most obvious being the dependence upon the quality of the data recorded in the medical records. Furthermore, a selection bias cannot be excluded. In fact, our hospital is the biggist orthopaedic hospital in Hebei Province. It was unavoidable to incorporate relatively more acute and severe patients in this study. What’s more, fog may influence cervical spine injury caused by traffic accidents to some degree, decreasing the credibility of the impact of snowing.

## Conclusions

The elder male patients and patients with head trauma are high-risk population for cervical cord injury. As a seasonal factor, snowing during heating season is of note a risk factor for cervical spine injury resulting from severe fall (>2 m) in the patients with cervical trauma in North China.
